# Therapeutic effects of pyrrolidine dithiocarbamate on acute lung injury in rabbits

**DOI:** 10.1186/1479-5876-9-61

**Published:** 2011-05-13

**Authors:** Meitang Wang, Tao Liu, Dian Wang, Yonghua Zheng, Xiangdong Wang, Jian He

**Affiliations:** 1 Department of Emergency Medicine, The Second Military University Changhai Hospital, China; 2 Department of Respiratory Medicine and Biomedical Research Center, Fudan University Zhongshan Hospital, Shanghai, China

**Keywords:** acute lung injury, TNF-α, ICAM-1, NF-κB, pyrrolidine dithiocarbamate

## Abstract

**Background:**

Acute lung injury (ALI) and acute respiratory distress syndrome (ARDS) is an early characteristic of multiple organ dysfunction, responsible for high mortality and poor prognosis in patients. The present study aims to evaluate therapeutic effects and mechanisms of pyrrolidine dithiocarbamate (PDTC) on ALI.

**Methods:**

Alveolar-arterial oxygen difference, lung tissue edema and compromise, NF-κB activation in polymorphonuclear neutrophil (PMN), and systemic levels of tumor necrosis factor-alpha (TNFa) and intercellular adhesion molecule-1 (ICAM-1) in rabbits induced by the intravenous administration of lipopolysaccharide (LPS) and treated with PDTC. Production of TNFa and IL-8, activation of Cathepsin G, and PMNs adhesion were also measured.

**Results:**

The intravenous administration of PDTC had partial therapeutic effects on endotoxemia-induced lung tissue edema and damage, neutrophil influx to the lung, alveolar-capillary barrier dysfunction, and high systemic levels of TNFa and ICAM-1 as well as over-activation of NF-κB. PDTC could directly and partially inhibit LPS-induced TNFa hyper-production and over-activities of Cathepsin G. Such inhibitory effects of PDTC were related to the various stimuli and enhanced through combination with PI3K inhibitor.

**Conclusion:**

NF-κB signal pathway could be one of targeting molecules and the combination with other signal pathway inhibitors may be an alternative of therapeutic strategies for ALI/ARDS.

## Background

Acute lung injury (ALI) and acute respiratory distress syndrome (ARDS) is an early characteristic of multiple organ dysfunction, which is responsible for high mortality and poor prognosis in patients with trauma, infection, shock, acute pancreatitis or sepsis [1]. Lipopolysaccharide (LPS) as the bacterial pathogen could trigger the over-production and over-expression of inflammatory mediators, including cytokines, chemokines, adhesion molecules, reactive oxygen species, and reactive nitrogen species [2], Primary and/or secondary excessive production of those mediators could lead to the development of systemic inflammation and lung tissue damage as well as coagulation/anti-coagulation imbalance, endothelial barrier dysfunction, and multiple organ dysfunction [3]. ALI could result from the activation of cytokine networks and the induction of proinflammatory gene expression, mediated by activating an inducible transcription factor, such as nuclear factor-κB (NF-κB), a driving force in the initiation and progression of systemic inflammation, ALI and multiple organ dysfunction [4,5].

The present study is aimed at evaluating the effects of pyrrolidine dithiocarbamate (PDTC), an inhibitor of NF-κB, on alveolar-capillary barrier dysfunction, lung tissue edema and compromise, NF-κB activation in polymorphonuclear neutrophil (PMN), and systemic levels of tumor necrosis factor-alpha (TNF-α) and intercellular adhesion molecule-1 (ICAM-1) in rabbits induced by the intravenous administration of lipopolysaccharide (LPS). Furthermore, direct effects of PDTC and dexamethasone (DEX) used as reference on PMN activities characterized by the production of TNF-α and cell activation of Cathepsin G were also studied. We also investigated the potential variation of PDTC effects on PMNs adhesion after different stimulations with leukotriene-B4 (LTB4), interleukin-8 (IL-8), and LPS and compare the therapeutic effects of the combination of PDTC and wortmannin.

## Materials and methods

### Induction of ALI

New Zealand rabbits with a mixture of female and male, weighing 2.0 kg, were used. The rabbits were kept in a 12:12-h night-day rhythm, fed with standard chow, and provided water *ad libitum*. The study was approved by the Animal Care Committee of The Second Military University and performed in accordance with the Guide for the Care and Use of Laboratory Animals. The rabbits were anesthetized with intravenous injection of 20% urethane at the dose of 5 ml/Kg. The femoral vein and homo-lateral femoral artery were separated, exposed and cannulated with a heparinized pediatric cardiac catheter for fluid replacement, drug delivery and blood sampling, respectively. Endotoxemia-associated ALI was induced by an intravenous injection of LPS (*Escherichia coli*, O111:B4, L-2630, Sigma Chemical, St. Louis, MO) at the dose of 500 μg/kg. Vehicle or PDTC at the dose of 100 mg/kg PDTC (Sigma) was intravenously administered one hour after the induction. Ringer's solution was intravenously infused continuously at the speed of 8 ml/kg/h during the experiment.

### Sampling

Blood was sampled before LPS injection as 0 h, and then 1, 2, 4 and 6 hours after LPS injection, respectively, for the measurement of arterial blood gas analysis. Blood was collected and centrifuged at 3000 × g for 5 min and the serum was stored at -80°C for the measurements of TNF-α and ICAM-1 assay and isolation of PMNs. The same volume of fluid was replaced in all animals after sampling. The superior lobe and inferior part of the right lung was harvested for measurement of dry/wet (D/W) ratio and pathology, respectively. The lung tissue was cleansed of blood and weighed as wet weight, and then kept a 75°C for 72 h for dry weight to calculate the lung D/W weight ratio.

### Pathological score

The lung was perfused through the bronchus at 20 cmH_2_O and fixed with 10% formaldehyde solution after the experiment was terminated. The lung tissues were embedded in paraffin wax, stained with hematoxylin and eosin, and examined under a light microscope. The lung injury was scored according to inflammatory changes, hemorrhage of alveoli and interstitial tissue, and pulmonary edema. Each pathological change was scored on a scale from 0-3 (normal, 0; minimal change, 1; medium change, 2; and severe change, 3), as described previously [6].

### Alveolar-arterial oxygen difference

PaO_2_, PaCO_2_, and pH were measured by blood gas analyzer (ABL 111, Radiometer, Copenhagen, Denmark). PaO_2 _(alveolar oxygen tension) was calculated by the following equation. P_A_O_2 _= (barometric pressure - 47) × FiO_2 _- PaCO_2_R. R, an exchange ratio, is assumed as 0.8 as described previously [7]. The alveolar-arterial PO_2 _difference (P_A-a_O_2_) = (barometric pressure - 47) × FiO_2 _- PaCO_2_R - PaO_2_. The severity of gas exchange impairment (P_A-a_O_2_) was examined using the linear correlation coefficient.

### PMN isolation

PMNs were separated as described previously [8]. Briefly, neutrophils were purified under endotoxin-free conditions. Anti-coagulated blood was added to 6% dextran (mol wt 70,000) in 0.9% sodium chloride solution in a 3:1 ratio (vol/vol, blood/dextran) and kept at room temperature for 30 min. The leukocytes were aspirated and centrifuged at 1000 × g for 6 min and the pellet was then resuspended in 2 ml RPMI 1640 (GIBCO, New York) and underlaid with 42% Percoll (Pharmacia, New Jersey), followed by 51% Percoll, and centrifuged for 10 minutes at 275 × g. The cells were then washed twice in RPMI-1640, afterwards the erythrocytes were lysed. The final cell population was > 98% PMNs by differential staining and > 99% viable by trypan blue exclusion. Purified neutrophils were resuspended in RPMI 1640 supplemented at a final concentration of 5 × 10^6 ^cells/ml and incubated in 48-well cell culture plates at 37°C in a 5% CO_2 _humidified atmosphere.

### Nuclear protein extraction

Nuclear protein was extracted as described previously [4]. Briefly, PMN (5 × 10^6^) were lysed in the buffer containing HEPES (10 mM, pH 7.9), KCl (10 mM), EDTA (0.1 mM), dithiothreitol (1 mM, DTT), and phenylmethylsulfonyl fluoride (1 mM, PMSF). Proteins were protected with 1% protease inhibitor cocktail, containing antipain, aprotinin and leupeptin (500 μg, respectively), pepstatin (50 μg), bestatin (750 μg), phosphoramidone (400 μg), and trypsin inhibitor (500 μg, ROCHE, Mannheim, Germany) in 1 ml. The cell suspension was then centrifuged at 12000 × g for 5 min (4°C). The nuclear pellet was resuspended and rocked vigorously for 20 min and total protein concentration was determined by Bradford assay (Coomassie Plus, Pierce, Rockford, IL, USA).

### Electrophoretic mobility shift assay (EMSA)

Detection of DNA-protein binding by EMSA was done using LightShift chemiluminescent electrophoretic mobility shift assay kit (Pierce Biotechnology, Rockford, IL, USA). Binding reactions were performed by adding 2 μg of the nuclear extracts to a mixture containing 40 mol of biotin-labeled, double-stranded probes (5'-AGTTGAGGGGACTTTCCCAGGC-3') 7 in 20 μl of binding buffer [10 mM Tris (pH 7.5), 10 mM EDTA, 0.5 mM DTT, 50 mM NaCl, and 5% glycerol] containing 2 μg of poly(dI-dC):poly(dI-dC). For supershift experiments, antibody (1 μg) were added to aliquots of extract and incubated for 20 min on ice before the adding of the reaction mixture. Competition reaction mixtures contained a 100-fold molar excess of non-labeled double-stranded oligoDNAs. The mixtures were then resolved by PAGE and visualized by horseradish peroxidase-conjugated streptavidin.

### Measurements of TNF, ICAM-1 and IL-8

Levels of TNF, ICAM-1 and IL-8 in serum or cell supernatants were determined using enzyme-linked immunosorbent assay (ELISA) in accordance with the protocol provided by the manufacturer (LIFEKEY BioMeditech Co., American). Briefly, primary antibody was plated and incubated at room temperature overnight. Samples were added and incubated for 2 h, the plates were washed, and a biotinylated secondary antibody was added and incubated for 2 h. Plates were washed again, and streptavidin bound to horseradish peroxidase was added for 20 min. After a further wash, tetramethylbenzidine was added for color development, and the reaction was terminated with 2 M H_2_SO_4_. Absorbance was measured at 450 nm.

### Cathepsin G activity

Cathepsin G was isolated and the activity of Cathepsin G was measured as described previously [9,10]. In brief, neutrophils were suspended in PBS, sonicated trice and centrifugated at 600 × g for 10 min. The supernatant was centrifuged at 16,000 × g for 30 min and the pellet was resuspended in 1 M NaCl with 0.005% Triton X-100. Proteins were precipitated by ammonium sulfate (60% saturation) and then resuspended in 40 ml of 0.05 M Tris-HCl at pH 8.0. After the centrifugation, the supernatant was subjected to an elastin-Sepharose affinity chromatography column (2.5 × 20 cm) and equilibrated with 0.05 M Tris buffer at pH 8.0. The part of cathepsin G was eluted with 1 M NaCl with 0.05 M Na acetate and 20% DMSO at pH 5.0, pooled and dialyzed in Vivaspin cut-off columns (5000 MWCO) in 1 M NaCl with 20 mM Na acetate at pH 5.5. It was then subjected to ion-exchange chromatography (CM Sephadex C-50) column and washed thrice, and the bound material was eluted by a linear NaCl gradient from 0.15 to 1 M. 5 ml was collected at a flow rate of 30 ml/h. Purified enzyme (0.2 μg) was diluted in 200 μl of HEPES 0.1 M, NaCl 0.5 M (pH 7.4) and 10% DMSO, and incubated with N-Suc-Ala-Ala-Pro-Phe-pNA (Suc-AAPF-pNA, 1 mM) as substrate. The absorbance was measured at 410 nm at 25°C.

### PMN adhesion

Neutrophils from normal rabbits were isolated, purified and cultured. Neutrophil adhesion was measured with a slight modification of the previous demonstration [11]. Cells were labeled with 2', 7'-bis(2-carboxyethyl) -5(6)-carboxyfluorescein acethoxymethyl ester (BCECF/AM, 10 μg/mL; Sigma, MO) for 30 min at 37°C. RPMI-1640 containing 2% fetal calf serum was added for the terminal reaction. Human umbilical vein endothelial cells (HUVECs) and endothelial cell growth medium (EGM-2, CC3156) were purchased (Clonetics, San Diego, CA), containing 10% fetal bovine serum, hydrocortisone, hFGF-B, vEGF, R3-IGF-I, ascorbic acid, hEGF, GA-1000, and heparin. HUVECs were cultured in 24-well plates until confluent, at which time different concentrations of SHBM1009 were added and then incubated for an additional 12 hours. KC and LTB4 (10 ng/mL) was added to the wells and incubated for 24 hours and HUVECs were then co-incubated with 10^6 ^labeled neutrophils/well for 30 minutes at 37°C. After removing non-adhering cells and washing and lysing adhering cells, fluorescence was measured with an excitation at 510 nm and emission at 550 nm. The increasing adhesion rate was calculated with the following formulation: [fluorescence intensity in stimulating cells - fluorescence intensity in non-stimulating cells]/fluorescence intensity in stimulating cells X 100.

### Experimental design

In order to evaluate the concept of therapeutic effects of NF-κB inhibitor, 60 rabbits were randomly allocated into three groups (n = 20): 1) animals were challenged and treated with vehicle (Group A), 2) animals were challenged with LPS and treated with vehicle (Group B) and 3) animals were challenged with LPS and treated with PDTC (Group C). The ALI was defined by measuring lung tissue edema (dry/wet weight ratio), lung damage (pathology) and dysfunction (P_A-a_O_2_). Systemic inflammatory response was monitored by the serum levels of TNF, IL-8 and ICAM-1, whereas NF-κB involvement was indicated by PMN NF-κB activities. In order to understand the direct effect of PDTC on PMNs, after the cells reached confluence, PMNs (10^6^) were treated with vehicle, PDTC (100 nM) or dexamethesone (DEX) dissolved in dimethyl sulfoxide (final 0.1%) for 4 h in serum-free RPMI medium and challenged with vehicle or LPS at 1 μg/ml for 24 hours.

Dose-associated effects of PDTC on different stimuli-induced PMN activation was monitored by measuring PMN adhesion 24 hours after the stimulation with vehicle, LPS, IL-8 and leukotriene B4 (LTB4) at 1 μg/ml. In order to evaluate the potential involvement of phosphoinositide 3-kinase (PI3K) in the activity of PMNs, cells were treated with vehicle, wortmannin (WT, a specific, covalent and irreversible inhibitor of the class I, II, and III PI3K members, 100 nM), PDTC (100 nM), or combination of WT and PDTC and IL-8 production was measured.

### Statistic analysis

Data were expressed as means ± standard deviations. The data from female and male rabbits were pooled after there was no statistical significance between them. Groups were compared by Repeated Measures Analysis of Variance and Kruskal-Wallis test. Least Significant Difference (LSD) test and the *Nemenyi *test were used for comparison between two groups. The statistical analysis was conducted by SAS 9.1.3 software. *P *value less than 0.05 is considered as significant.

## Results

**Figure 1:**
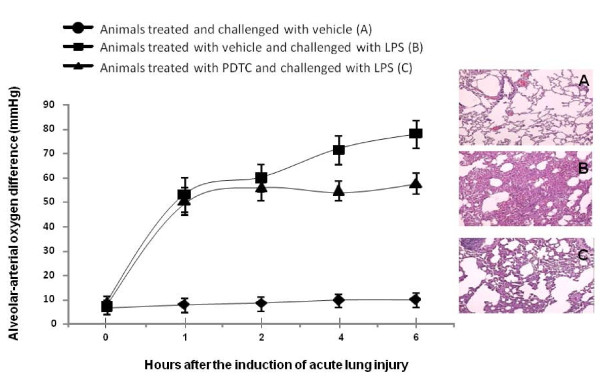
**Values of alveolar-capillary oxygen difference in animals**. Animals were treated and challenged with vehicle (A), treated with vehicle and challenged with lipopolysaccharide (LPS) (B), or treated with pyrrolidine dithiocarbamate (PDTC) and challenged with LPS (C). Animals were intravenously challenged and treated for 0 (before challenge), 1, 2, 4 and 6 hours and each group had 20 animals. Histological photographs of the lung (hematoxylin & eosin, X200) 6 hours after the intravenous challenge and treatment.

No animals died before the termination of experiment. The values of P_A-a_O_2 _in all animals treated with vehicle or PDTC from 1 hour and onwards after ALI induction were significantly higher, as compared with those treated and challenged with vehicle (Figure 1, p < 0.01, respectively). Values of ALI animals treated with PDTC were significantly higher than those with vehicle 4 and 6 hours after the administration of LPS (p < 0.05). Pathological alterations of ALI animals treated with vehicle or PDTC were showed in Figure 1. The lungs of animals treated with vehicle and challenged with LPS had thicker alveolar wall, infiltration of leukocytes of which more than 90% were neutrophils, intra-alveolar hemorrhage, formation of micro-thrombosis, alveolar deteleotasis and edematous fluid in alveolar space (Figure 1B). Pathological alterations in the lungs of animals with LPS and PDTC were less severe, including clearer alveolar structure and compromise as well as leukocyte influx (Figure 1C). There were still definite changes when compared with animals treated and challenged with vehicle (Figure 1A).

**Figure 2:**
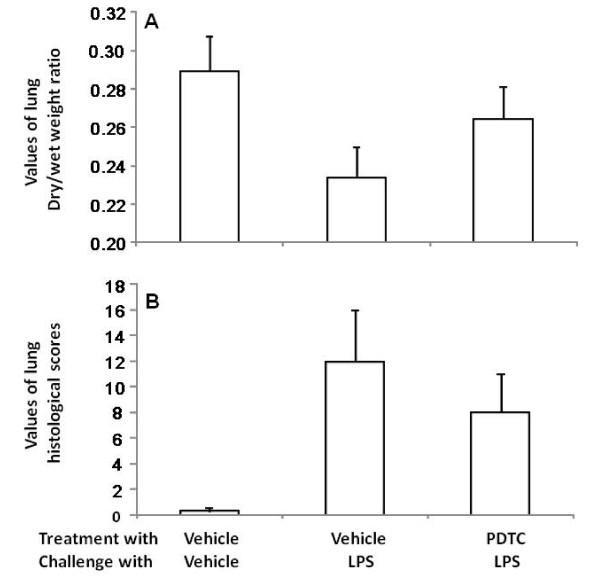
**Values of dry/wet lung weight and histological score in animals**. Animals were treated and challenged with vehicle (A), treated with vehicle and challenged with lipopolysaccharide (LPS) (B), or treated with pyrrolidine dithiocarbamate (PDTC) and challenged with LPS (C). Animals were intravenously challenged and treated for 0 (before challenge), 1, 2, 4 and 6 hours and each group had 20 animals.

Values of lung dry/wet weight of animals challenged with LPS and treated with vehicle or PDTC were significantly lower than those challenged and treated with vehicle (Figure 2A, p < 0.01 or 0.05, respectively). Animals treated with PDTC had significantly higher levels of lung dry/wet weight than those with vehicle 24 hours after the administration of LPS (p < 0.05). Histological scores of lung pathology in animals challenged with LPS and treated with vehicle or PDTC were significantly higher than those without LPS (Figure 2B, p < 0.01, respectively).

**Figure 3:**
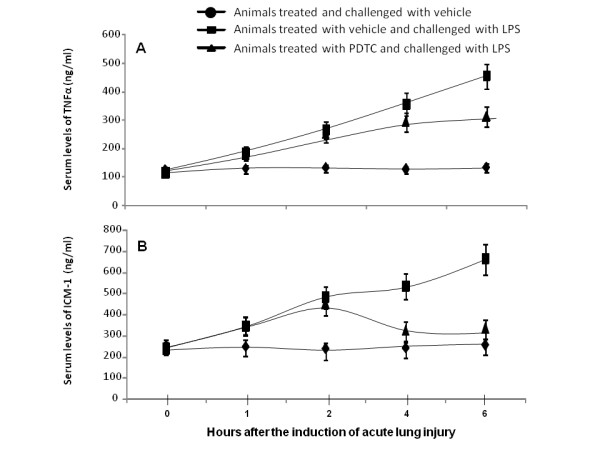
**Serum levels of tumor necrosis factor-alpha (TNF-α) and intercellular adhesion molecule-1 (ICAM-1) in animals**. Animals were treated and challenged with vehicle (A), treated with vehicle and challenged with lipopolysaccharide (LPS) (B), or treated with pyrrolidine dithiocarbamate (PDTC) and challenged with LPS (C). Animals were intravenously challenged and treated for 0 (before challenge), 1, 2, 4 and 6 hours and each group had 20 animals.

Serum levels of TNFα significantly increased in animals treated with vehicle or PDTC from 1 hour after LPS injection, as compared to those challenged with vehicle (Figure 3A, p < 0.01, respectively). Animals treated with PDTC had significantly lower serum levels of TNFα than those with vehicle 4 and 6 hours after LPS challenge (p < 0.05). Serum levels of ICAM-1 in animals treated with vehicle were significantly higher than both those with PDTC 4 and 6 hours after LPS challenge or those challenged and treated with vehicle (Figure 3B, p < 0.01, respectively). However, animals challenged with LPS and treated with vehicle or PDTC has significantly higher levels of ICAM-1 than those treated and challenged with vehicle at 1 and 2 hours (p < 0.05).

**Figure 4:**
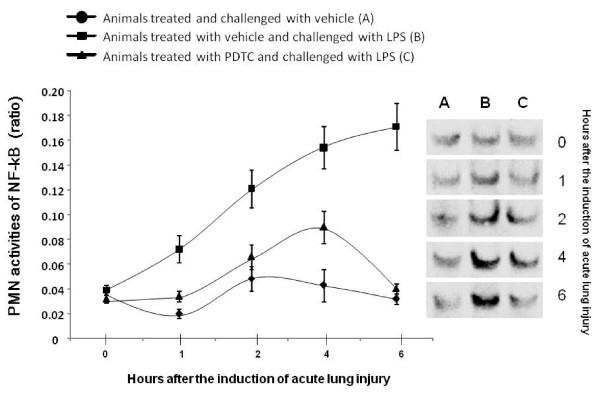
**Activities of nuclear factor kappa B (NF-κB) in polymorphonuclear neutrophils (PMN)**. Activities were calculated as referred to the average value of PMN NF-κB activities before the intravenous challenge and treatment. PMNs were isolated from animals treated and challenged with vehicle (A), treated with vehicle and challenged with lipopolysaccharide (LPS) (B), or treated with pyrrolidine dithiocarbamate (PDTC) and challenged with LPS. Animals were intravenously challenged and treated for 0 (before challenge), 1, 2, 4 and 6 hours and each group had 20 animals. Representatives of the electrophoretic mobility shift assay of NF-κB activation in PMN were also shown.

Fig 4 demonstrates the ratio of NF-κB activity between the densities of each measurement with the mean value at 0 hour and representative results of EMSA analyses of NF-κB activation in PMNs (Figure 4A-C). NF-κB activity in PMNs from animals treated with vehicle significantly increased from 1 after LPS challenge, as compared with those treated with PDTC or without LPS (p < 0.05 or 0.01, respectively). There was no statistical difference of NF-κB activity between animals with LPS and PDTC or without LPS, except for that at post-challenge 4 hours (p < 0.05, Figure 4).

**Figure 5:**
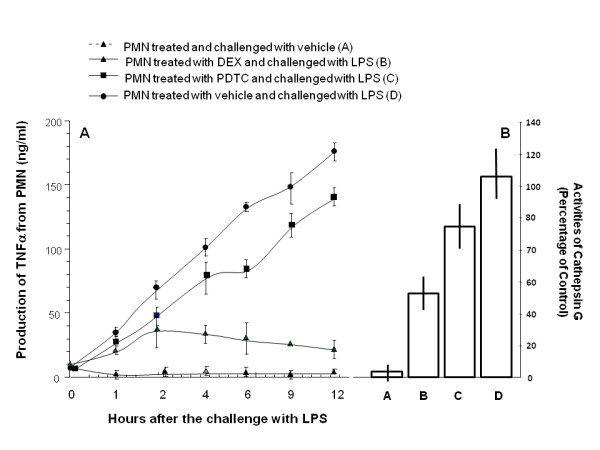
**Levels of tumor necrosis factor-alpha (TNF-α) in the supernatant of cell culture and activities of Cathepsin G of polymorphonuclear neutrophils (PMN)**. Cells were treated and challenged with vehicle (A), treated with dexamethasone (Dx) and challenged with lipopolysaccharide (LPS) (B), treated with pyrrolidine dithiocarbamate (PDTC) and challenged with LPS (C), or treated with vehicle and challenged with LPS (D). The levels of TNF-a were measured 0, 1, 2, 4, 6, 9 and 12 hours after treatment and challenge, while activities of Cathepsin G in PMNs were measured 12 hours after treatment and challenge.

In order to evaluate direct effects of LPS on PMNs, PMNs were stimulated directly by LPS during cell culture and activities of PMNs were indicated by production of TNFα and cathepsin G. The production of TNFα from LPS-stimulated cells treated with vehicle, PDTC or DEX significantly increased with time, as compared with those without LPS (Figure 5A, p < 0.05 or 0.01, respectively). Levels of TNFα from LPS-stimulated PMNs treated with PDTC or DEX were significantly lower than those treated with vehicle (p < 0.05 or 0.01, respectively). There was also significant difference between LPS-stimulated cells with PDTC or DEX (p < 0.05 or 0.01, respectively). LPS-stimulated cells had significantly higher activity of Cathepsin G than cells with LPS, while PDTC and DEX significantly reduced LPS-induced over-activity and DEX showed even better results than PDTC (p < 0.05, respectively, Figure 5B).

**Figure 6:**
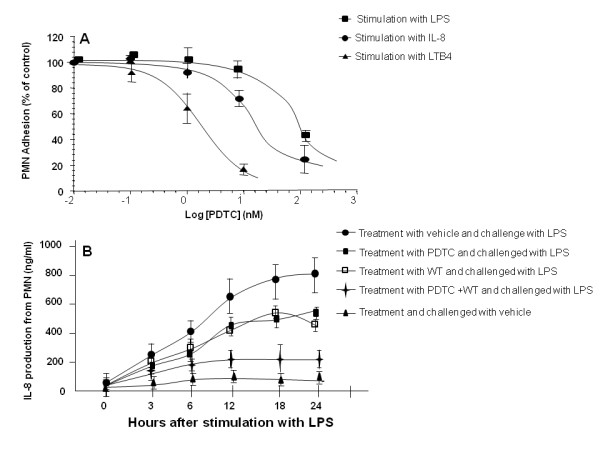
**The adhesion of polymorphonuclear neutrophils (PMN)**. The adhesion was measured 24 hours after treatment with pyrrolidine dithiocarbamate (PDTC) at different concentrations and challenges with leukotriene B4 (LTB4), interleukin-8 (IL-8) and lipopolysaccharide (LPS). Levels of IL-8 in the supernatant of PMN culture were measured 0, 3, 6, 9, 12, 18 and 24 hours after the challenge with LPS or vehicle and treatment with vehicle, PDTC alone, wortmannin (WT) alone or the combination of PDTC and WT.

PDTC showed significant inhibitory effects on PMN adhesion induced by LTB4, IL8 and LPS at different doses, as shown in Figure 6A. Of them, LTB4-stimulated cell adhesion was more sensitive to PDTC than IL-8 and LPS, and IL-8-stimulated adhesion was more sensitive than LPS did (p < 0.05). Cells treated with WT or PDTC had significantly lower IL-8 production than those with vehicle after LPS challenge (Figure 6B, p < 0.05 or 0.01, respectively), even though those productions were still significantly higher than cells without LPS challenge (p < 0.01, respectively). The production of IL-8 from cells treated with the combination of WT and PDTC was significantly lower than that from cells with WT or PDTC alone (p < 0.01, respectively).

## Discussion

Endotoxemia often happens due to the primary infection or secondary gut origin sepsis [12-15], leading to the development of ALI in the early stage of diseases [16-18]. Multiple intracellular signal pathways, cellular receptors, inflammatory mediators, cells and systems have been suggested as contributors to the pathogenesis of ALI/ARDS. Of them, NF-κB was proposed to be the central and critical factor, regulating the production of inflammatory mediators [18]. NF-κB inhibitor could attenuate endotoxin-induced ALI [19]. Most of those investigations were performed in mice and rats, which have their own advantages and limits, especially for the evaluation of drug efficacy [2]. The present study was performed in rabbits and found that PDTC had partial therapeutic effects on endotoxemia-induced ALI.

Those partial effects of PDTC included were found on endotoxemia- induced dysfunction of oxygen exchange between alveolar-capillary barrier, neutrophil influx to lung tissue, and lung edema and damage. The reason why our data did not show the fully inhibitory effects of PDTC on ALI as others found [19,20] may be due to that PDTC was administered after LPS challenge as the therapeutic process to mimic the situation in clinic. It is also possible that PDTC has different effects between small and large animals, or that the severity of ALI in our model was more serious. Endotoxins trigger the production of inflammatory cytokines, responsible for lung compromise and multiple organ failure [21]. Our results demonstrated that PDTC could partially inhibit the production of TNF-α while having more effects on the production of ICAM-1, even though both may play critical roles in endotoxin-induced inflammatory response [22] and were considered as markers of NF-κB activation [19]. However, the previous study demonstrated that the pretreatment with PDTC did not affect TNF-α production in bronchoalveolar lavage fluid, mRNA expression of TNF-α and ICAM-1 in the lung tissue or NF-κB activation in macrophages and neutrophil oxidant production [19].

Neutrophils and their production of inflammatory cytokines, oxygen free radicals, and enzymes together play the important role in the pathogenesis of ALI. Our previous studies showed that neutrophils made up more than 95% of total leukocytes infiltrated into either the lung tissue or alveolar space in mice with LPS-induced ALI [23]. In the present study, we also noticed that the neutrophil influx into the lung tissue increased in rabbits with endotoxemia-induced ALI, while being partially inhibited by PDTC. However, other studies demonstrated that PDTC prevented primary or secondary ALI induced by LPS or mesenteric ischemia/reperfusion probably due to the inhibitory effects on lung lipid peroxidation, malondialdehyde, glutathione, and nitric oxide, rather than the reduction of pulmonary neutrophil sequestration and oxidant production [19,24]. Our study showed evidence that PDTC could directly inhibit the activation of PMNs characterized by the production of TNF-α and the activity of Cathepsin G.

Inhibitory effects of PDTC were dependent upon the stimuli, supported by the fact that LPS-stimulated cell adhesion had less sensitive to PDTC than LTB4 and IL-8. LTB4 induced a rapid but transient adhesion of PMN to an albumin-coated plastic surface and to cultured human umbilical vein endothelial cells associated with leukocyte adhesion protein CD18 [25]. IL-8 is one of the most chemoattractant factors causing PMN adhesion and migration, probably through the phosphorylation and translocation of cytosolic gIVaPLA2 to the nucleus, change in cell shape, polymerization of F-actin, tyrosine phosphorylation as well as enzymatic activity of proline-rich tyrosine kinase 2, a non-receptor protein tyrosine kinase [26,27]. The PMN response to LPS was less sensitive in the absence of serum, since LPS stimulated neutrophils by interacting with specific cellular receptors, although upregulation of CD11b/CD18 could still be seen using higher concentrations of LPS [28]. Our data also indicate that LPL-stimulated response had less sensitivity to PDTC which may contribute to the partial inhibitory effects of PDCT.

Activities of NF-κB were increased and associated with the levels of inflammatory mediators in BAL fluid from patients with ARDS [29,30]. In addition, NF-κB activation has been identified in alveolar macrophages from humans with ARDS [31]. Endotoxins can activate NF-κB and then initiate transcription and interpretation of many cytokine genes [32,33] closely related with inflammation and immune reaction. NF-κB plays a critical role in the transcriptional activation of multiple genes that contributed to the development of ALI [34]. The present study showed that NF-κB activity in PMNs increased, accompanied with elevated levels of TNF-α and ICAM-1 in the early stage of ALI, while PDTC could reduce LPS-induced over-activation of NF-κB. Although it should be stated that PDTC has been considered as the NF-κB inhibitor, but it also has another multitude of effects, e.g. antioxidant [19,35]. For example, the protective effects of PDTC on LPS-induced ALI was proposed to be associated with antioxidant rather than NF-κB activity, since pre-treatment with PDTC failed to reduce on LPS-induced NF-κB DNA binding activity in macrophage nuclear extracts [19]. The present study showed the therapeutic effects of PDTC on over-activation of NF-κB in neutrophils. However, the down-regulated activities of NF-κB did not show a clear correlation and consistency with the therapeutic effects of PDTC on systemic levels of TNF-α, lung tissue edema and damage, and lung dysfunction induced by LPS.

It was hypothesized that PDTC may interfere with NF-κB DNA binding activity through phorbol ester 12-O-tetradecanoylphorbol-13-acetate (TPA) or TNF-α-stimulated signaling pathway. PDTC did not inhibit TNF-α-induced NF-kappaB DNA binding activity but potentiated the effect of TNF-α on kappaB-dependent gene expression. PDTC could induce AP-1 DNA binding and AP-1 reporter gene activity, leading to the inhibition of NF-κB activity [36]. TPA-induced signaling pathway includes the activation of extracellular signal-regulated kinase 1/2, p38 mitogen-activated protein kinase (MAPK), and PI3K/Akt, which are upstream of NFκB. Our data showed that the combination of PDTC with PI3K inhibitor Wortmannin had more inhibitory effects on LPS-induced PMN overproduction of IL-8, than either on its own. Wortmannin is a specific, covalent inhibitor of PI3Ks, for the class I, II, and III PI3K members, although it can also inhibit other PI3K-related enzymes such as mTOR, DNA-PK, some PI4Ks, myosin light chain kinase, members of the polo-like kinase family and MAPK [37,38]. It indicates that multiple signaling pathways associated PI3K-NF-κB communication may be involved in the hyper-activation of PMNs and endotoxemia-induced ALI. This was also supported by the finding that inhibitory effects of DEX on LPS-induced TNF-α production and Cathepsin G over-activation were significantly better than PDTC. It seems that the inhibitory effects of PDTC were not only dependent upon the variation of stimuli and severities of the disease, but also different between targeting cells. For example, effects of PDTC on macrophages might be related with the antioxidant process rather than TNF-α and NF-κB [19], but not on the epithelial cells [39]. However, this is the preliminary study to evaluate PDTC effects in large animals, so it would be important to show the dose-dependent efficacy of PDTC and additional target-specific inhibitors, even though it may be difficult to be found for rabbits. It is also more helpful if the study could measure the recruitment of leukocytes from the circulation to the interstitial tissue and alveolar space. The use together with blocking a PI3K imply potential effect in a multimodal therapeutic setting, which should be further explored due to the complexity of mechanisms involved in ALI.

## Conclusion

The present study demonstrated that the intravenous administration of PDTC had partial therapeutic effects on endotoxemia-induced lung tissue edema and damage, neutrophil influx to the lung, alveolar-capillary barrier dysfunction, and high systemic levels of TNF-α and ICAM-1 as well as over-activation of NF-κB. PDTC could directly and partially inhibit LPS-induced TNF-α hyper-production and over-activities of Cathepsin G. Such inhibitory effects of PDTC were related to the various stimuli and enhanced through combination with PI3K inhibitor. Thus, our data indicate that NF-κB signal pathway may be one of the molecules to target and the combination with other signal pathway inhibitors may be an alternative of therapeutic strategies for ALI/ARDS.

## Competing interests

The authors declare that they have no competing interests.

## Contributions

MTW: performing the study and data analysis and writing manuscript

TL: making study plan and performing the study anddata analysis

DW: make study plan and performing study, as well as editing manuscript

YHZ: performing study and editing manuscript

XDW: making study plan and advising data analysis as well as writing manuscript

JH: making study plan and advising data analysis as well as writing manuscript

All authors read and approved the final manuscript
